# Fluoroquinolone Metalloantibiotics to Bypass Antimicrobial Resistance Mechanisms: Decreased Permeation through Porins

**DOI:** 10.3390/membranes11010003

**Published:** 2020-12-22

**Authors:** Mariana Ferreira, Carla F. Sousa, Paula Gameiro

**Affiliations:** REQUIMTE-LAQV (Rede de Química e Tecnologia—Laboratório Associado para a Química Verde), Departamento de Química e Bioquímica, da Faculdade de Ciências, da Universidade do Porto, Rua do Campo Alegre, s/n, 4169-007 Porto, Portugal; cfilipams@gmail.com (C.F.S.); agsantos@fc.up.pt (P.G.)

**Keywords:** fluoroquinolones, metalloantibiotics, bacterial resistance, liposomes, porins, fluorescence, surface plasmon resonance

## Abstract

Fluoroquinolones (FQs) are broad-spectrum antibiotics largely used in the clinical practice against Gram-negative and some Gram-positive bacteria. Nevertheless, bacteria have developed several antimicrobial resistance mechanisms against such class of antibiotics. Ternary complexes of FQs, copper(II) and phenanthroline, known as metalloantibiotics, arise in an attempt to counteract an antibiotic resistance mechanism related to low membrane permeability. These metalloantibiotics seem to use an alternative influx route, independent of porins. The translocation pathways of five FQs and its metalloantibiotics were studied through biophysical experiments, allowing us to infer about the role of OmpF porin in the influx. The FQ-OmpF interaction was assessed in mimetic membrane systems differing on the lipidic composition, disclosing no interference of the lipidic composition. The drug-porin interaction revealed similar values for the association constants of FQs and metalloantibiotics with native OmpF. Therefore, OmpF mutants and specific quenchers were used to study the location-association relationship, comparing a free FQ and its metalloantibiotic. The free FQ revealed a specific association, with preference for residues on the centre of OmpF, while the metalloantibiotic showed a random interaction. Thereby, metalloantibiotics may be an alternative to pure FQs, being able to overcome some antimicrobial resistance mechanism of Gram-negative bacteria related to decreased membrane permeability.

## 1. Introduction

Together with epidemics, outbreaks of infectious diseases and environmental hazards antimicrobial resistance (AMR) is one of the major public health threats of the 21st century, [1,2]. Due to its critical impact in the public health and expensive healthcare costs, AMR has become a significant worldwide concern under systematic surveillance [3].

The first natural barrier of bacteria against antimicrobials is their cell envelope [4,5]. The membrane of Gram-negative bacteria is a complex structure, comprising two membranes, the inner membrane (cytoplasmic membrane) and the outer membrane [6]. The cytoplasmic membrane is a phospholip bilayer, enriched with zwitterionic and anionic phospholipids, especially PE (around 60–80%), PG (about 20%) and CL (up to 10%) [7,8,9]. In turn, the outer membrane is an asymmetric bilayer, whose leaflets (inner and outer) differ in composition. The inner leaflet is mostly composed by phospholipids, being extremely similar to the cytoplasmic membrane [10]. In contrast, the outer leaflet of the outer membrane is enriched with glycolipids, mainly lipopolysaccharides (LPS) [5,8,10,11]. Additionally, this leaflet also harbours lipoproteins (attached to lipids) and integral membrane proteins, known as outer membrane proteins (Omps) or porins [9,12]. These channels are responsible for the transport of nutrients and some antibiotics as FQs or β-lactams [11,13,14].

Fluoroquinolones (FQs) are a family of broad-spectrum antibiotics, active against Gram-negative and some Gram-positive bacteria, being widely used in the clinical practice [15]. These antibiotics act intracellularly, inhibiting the activity of DNA gyrase and topoisomerase IV [16,17], and their translocation into the Gram-negative bacterial cytoplasm is usually dependent on porins [18]. *Escherichia coli,* a well-characterized Gram-negative bacterial model, has two main porins responsible for the translocation of FQs, OmpF and OmpC [13,19]. Between these two porins, OmpF has proved to be the major channel implicated in the transport of several FQs in *E. coli* [19,20].

Bacteria have developed several resistance mechanisms to avoid the action of FQs. Alterations of the target molecules (through chromosomal mutations or plasmid-acquired resistance genes) and the reduction of the intracellular concentration of the drugs (by decreasing the influx or increasing the efflux) are the main mechanisms of resistance to FQs [21,22].

One of the strategies to bypass the bacterial resistance to FQs is their complexation with transition metals [23,24,25,26,27]. FQs have two ionizable groups of FQs, the carboxyl and the amino groups, responsible for their amphoteric character [28]. These antibiotics easily chelate to transition metals via the carboxyl group of the 4th position and the exocyclic oxygen of the 3rd position [23,29,30]. Over the last years several studies have been performed, evidencing that copper (II) complexes are very stable in solution [23,24,25,27]. Additionally, it was discovered that complexes of copper and 1,10-phenanthroline (phen), a nitrogen donor heterocyclic ligand, possess nuclease activity [31,32,33]. These findings gave rise to the synthesis and characterization of ternary complexes of copper (II), 1,10-phen and FQ (CuFQphen). These compounds, known as metalloantibiotics, are highly stable under physiological conditions, pH and temperature and exhibit antibacterial activity (comparable or improved to pure FQs) [23,30,34].

During the last years, several studies focusing on the translocation of FQs and their metalloantibiotics have been performed [14,20,23,35,36,37,38,39]. Generally, the behaviour adopted by pure FQs differs from the respective metalloantibiotic. Beginning with the analysis of the partition into the bacterial membrane, the partition constants (Kp) determined for the metalloantibiotics are usually higher compared to the ones of the free FQs [36,37,38,39], proposing a facilitated diffusion into the membranes. Concerning the role of porins for the influx, all free FQs, with exception of moxifloxacin (mxfx) and sparfloxacin (spx), evidenced the use of porins (especially OmpF and/or OmpC) to their uptake in microbiological tests [23]. At physiological pH (7.4), the carboxylic acid of FQs is deprotonated (pH > pKa1) and the amino group is commonly protonated (pH < pKa2), meaning that FQs exist mainly in a mixture of neutral (HFQ^0^) and zwitterionic forms (HFQ^±^) [40,41,42]. The zwitterionic form, predominant for the majority of FQs, has a high affinity to the electrostatic residues of the constriction zone of porins, favouring its transport through Omps [35,41,42]. In contrast to free FQs, the majority of the metalloantibiotics proved to penetrate into the bacterial cell in an independent-porin way [23]. These facts are corroborated by theoretical results evidencing decreased affinities of metalloantibiotics to the constriction zone of the porins, compared to free FQs. On the contrary, the interaction of the metalloantibiotics with OmpF proved to relay on residues located outside the constriction zone [35]. Analysing the high Kp values of metalloantibiotics and their speciation in solution, it is expected an influx route based on the passive diffusion across the membrane. All these findings support an influx route of metalloantibiotics alternative to that used by FQs, possibly overcoming some bacterial resistance mechanisms adopted against free FQs, thus being promising against resistant bacteria [23,25,30,43,44].

In this work, the translocation route of five FQs and their metalloantibiotics was studied, combining different biophysical approaches. The interaction of the compounds with OmpF protein was assessed in proteoliposomes mimicking the *E. coli* membrane by fluorescence spectroscopy and Surface Plasmon Resonance (SPR), through association and location studies. This study was performed for a broad set of compounds belonging to different generations of FQs—ciprofloxacin (cpx) and enrofloxacin (erx)—2nd generation; levofloxacin (lvx) and spx—3rd generation; mxfx—4th generation [23,25,28]—and using a bacterial mimetic system of excellence composed by proteoliposomes of OmpF/*E. coli* total lipidic extract. The results obtained show similar association constants for FQs and metalloantibiotics, determined by fluorescence spectroscopy and SPR. However, location studies using mutants of OmpF and specific quenchers revealed differences between a free FQ and its metalloantibiotic. The binding observed for the free FQ revealed to be specific, with preferential interaction in the centre of the porin, while its metalloantibiotic showed a random interaction with residues located in different regions of the porin. These results support different influx routes of free FQs and metalloantibiotics, as previously proposed by other authors [23,25,30,43,44], encouraging metalloantibiotics as promising alternatives to circumvent some AMR mechanisms based on changes of the membrane permeability.

## 2. Materials and Methods

### 2.1. Fluoroquinolones and Metalloantibiotics Preparation

All compounds were used as received. Ciprofloxacin (cpx), enrofloxacin (erx), levofloxacin (lvx) and sparfloxacin (spx) (all >98.0%), were purchased from Sigma-Aldrich (Saint Louis, MO, USA). Moxifloxacin (mxfx) was a gift from Bayer. Fluoroquinolones (FQs) were stored at room temperature (cpx, lvx, mxfx and spx) or at 4 °C (erx), protected from light. All solutions were prepared in 10 mmol dm^−3^ N-(2-hydroxyethyl) piperazine-*N*’-ethanesulfonic acid (HEPES, Sigma-Aldrich) buffer (0.1 mol dm^−3^ NaCl; pH 7.4, using double deionised water), with the exception of copper (Cu(NO_3_)_2_·3H_2_O salt, Merck, Darmstadt, Germany) solution that was prepared in double deionised water. The metalloantibiotic solutions used were prepared by mixing the FQ, Cu(II) and 1,10-phenanthroline (phen, Sigma-Aldrich) in stoichiometric proportions (1:1:1), as previously reported for other FQ metalloantibiotics [23,24,25,36,38,39]. The copper solution used to prepare the metalloantibiotics solutions was previously titrated in alkaline medium with ethylenediaminetetraacetic acid (EDTA, Sigma-Aldrich) and using murexide (Fluka, Charlotte, NC, USA) as indicator. The iodide solution used in the location studies was prepared with sodium thiosulfate (10 mmol dm^−3^) in order to avoid the rapid oxidation of potassium iodide solution. The quantification of iodide in the potassium iodide solution was determined in acid medium, with potassium iodate and using carbon tetrachloride as indicator. All compound solutions prepared were stored at 4 °C and protected from light.

### 2.2. Proteoliposome Preparation

OmpF proteoliposomes were prepared by protein incorporation into preformed liposomes. 1-palmitoyl-2-oleoyl-*sn*-glycero-3-phosphoethanolamine (POPE), 1-palmitoyl-2-oleoyl-*sn*-glycero-3-phospho-(1′-*rac*-glycerol) (POPG), cardiolipin (CL—natural extract from *Escherichia* *coli*) and *E. coli* total lipid extract (PE 57.5%; PG 15.1%; CL 9.8%; unknown lipids 17.6%) were from Avanti Polar Lipids (Sigma-Aldrich). OmpF (outer membrane protein F) was a gift from Dr. Mathias Winterhalter (Jacobs University, Bremen, Germany) and from Dr. Ricardo Franco (Universidade Nova de Lisboa, Portugal). OmpF mutants (W61F and W214F) were also a gift from Dr. Mathias Winterhalter.

#### 2.2.1. Liposome Preparation

Liposomes were prepared by dryness of chloroform (Sigma-Aldrich) solutions containing the appropriate amount of the several used lipids (*E. coli* total lipid extract and POPE/POPG and POPE/POPG/CL mixtures), under a stream or argon. The lipid films were evaporated under vacuum for, at least, 3 h. Multilamellar vesicles (MLVs) were obtained by redispersion of the lipidic film in 10 mmol dm^−3^ HEPES buffer (0.1 mol dm^−3^ NaCl; pH 7.4). The suspensions were vortexed and submitted to five cycles of frozen/thawed, using liquid nitrogen and a water bath with a temperature above the phase transition temperature of each lipidic system. Large unilamellar vesicles (LUVs) were obtained by 10 times extrusion of MLVs, through 100 nm polycarbonate filters (Whatman, Maidstone, UK) on a Lipex Biomembrane extruder attached to a water bath. The extrusion was performed above the phase transition temperature of each system.

The phospholipid concentration in the liposome and proteoliposome suspensions was determined by phosphate analysis through a modified Bartlett method [45,46]. Briefly, the destruction of the phospholipids to inorganic phosphate was performed by incubation of the samples with perchloric acid 70% *v/v* (HClO_4_), for one hour, in a sand bath with controlled temperature between 180 °C and 200 °C. Parallel to the samples, a blank containing water and a set of standard solutions of potassium dihydrogen phosphate (concentrations between 0.05 and 0.3 mmol dm^−3^) were prepared. After cooling to room temperature, ammonium molybdate and Fiske-Subbarow reagent were added and the samples were incubated for seven minutes, at 100 °C. The amount of phosphate was determined by absorbance readings at 830 nm. All samples were prepared in replicates.

#### 2.2.2. OmpF Reconstitution into Liposomes

OmpF proteoliposomes were prepared by protein incorporation into preformed liposomes, according to Lopes et al. [47]. Briefly, protein reconstitution was performed by addition of OmpF solution containing 1% of octylpolyoxyethylene (o-POE, Bachem, Bubendorf, Switzerland) and o-POE (with a final concentration of detergent lesser than the micellar critic concentration of each system—0.26% *v*/*v* for POPE/POPG and *E. coli* total extract and 0.12% *v*/*v* for POPE/POPG/CL) to a LUVs suspension previously prepared. The mixture was incubated under gentle agitation for 30 min, at room temperature, followed by 1 h on ice. The excess of detergent was then removed by adsorption on polystyrene beads, incubating the mixture twice with Bio-Beads SM-2 (Bio-Rad, Hercules, CA, USA), using 75 mg per ml of the prepared proteoliposome. The first addition was performed for 3 h, at room temperature, and the second overnight, at 4 °C. The proteoliposome suspension was then submitted to cycles of frozen/thawed and extrusion, like previously described for liposomes. Again, the extrusion was realized above the phase transition temperature of each lipidic system. The proteoliposomes were prepared with a final protein/lipid molar ratio of 1:1000. During all the process, the size distribution of the particles was determined by dynamic light scattering (DLS) on a Zeta Sizer Nano Zs (Malvern Instruments, Malvern, UK), at 37.0 ± 0.1 °C. The final mean particle size was ≈100 nm for liposomes and ≈ 150 nm for proteoliposomes (polydispersity index < 0.1).

The protein concentration of OmpF was estimated using the bicinchoninic acid protein assay against bovine serum albumin (BSA) as standard [48,49]. The protein concentration was assessed for all received proteins and all prepared proteoliposomes.

### 2.3. Drug-Protein Interaction

#### 2.3.1. Fluorescence Studies

Steady-state fluorescence measurements were performed on a Varian Cary Eclipse spectrofluorometer equipped with a “Single Cell Peltier Accessory” temperature controller, in 1 cm quartz cuvettes. Concentrations of pure FQs and metalloantibiotics used in the fluorescence studies were chosen according to their Lambert-Beer law (absorbance value <0.1 at the excitation and emission wavelengths), to avoid the inner filter effect [50,51]. Absorption spectra were carried out on a UV-Vis-NIR (UV-3600) spectrophotometer (Shimadzu, Kyoto, Japan) equipped with a temperature controller (Shimadzu TCC-CONTROLLER). Spectra were recorded at 37.0 ± 0.1 °C, in 1 cm quartz cuvettes, with a slit width of 5 nm, in a wavelength range from 225 to 450 nm.

The OmpF association studies were performed by fluorescence spectroscopy, based on the intrinsic fluorescence from six Trp residues [20,52]. The OmpF-cpx association was assessed in POPE/POPG, POPE/POPG/CL and *E. coli* total extract proteoliposomes of native protein (OmpF WT). The interaction of pure FQs (cpx, erx, lvx and mxfx) and their metalloantibiotics was also determined in *E. coli* total extract proteoliposomes of native OmpF. Small aliquots (µL) of compound solution were successively added to an OmpF proteoliposome suspension of 1 mmol dm^−3^/1 µmol dm^−3^ (lipid/protein). After each addition, the mixture was equilibrated for five minutes and the spectra were traced, at 37.0 ± 0.1 °C, under constant stirring. The interaction between cpx and OmpF was determined using a drug maximum concentration of 18 µmol dm^−3^. In turn, the Cucpxphen/OmpF (WT and mutants) interaction was studied with a maximum compound concentration of 5 µmol dm^−3^. The maximum compound concentration used in each assay was 18 µmol dm^−3^ for cpx, 14 µmol dm^−3^ for erx, 6 µmol dm^−3^ for Cuspxphen, 5 µmol dm^−3^ for lvx and Cucpxphen, 4 µmol dm^−3^ for spx, Cuerxphen and Cumxfxphen and 3 µmol dm^−3^ for mxfx and Culvxphen. The spectra were recorded with an excitation wavelength of 290 nm and emission wavelength range from 300 to 380 nm, an excitation slit width of 5 nm and an emission slit width of 10 nm, scan rate of 120 nm/minute and data range of 1 nm. All fluorescence experimental data were corrected for the dilution effect [53] and three independent measurements were performed.

##### Fluorescence Data Analysis

The association of the studied compounds with OmpF resulted in the decrease of the fluorescence of the Trp residues. This process arises from a phenomenon of static fluorescence, as previous reported for the interaction of FQs with OmpF protein [54,55]. This process consists in the formation of a non-fluorescent complex between the quencher and the fluorophore in the ground-state. In this case, the constant can be considered as the association constant ($K_{ass}$) for the complex formation:

(1) $$K_{ass}=\frac{\left[F-Q\right]}{\left[F\right]\left[Q\right]}$$

where [F−Q], [F] and [Q] are the concentrations of the complex, of the uncomplexed fluorophore and of the quencher, respectively. As the total concentration of the fluorophore ${\left[F\right]}_{0}$ is given by:

(2) $${\left[F\right]}_{0}=\left[F\right]+\left[F-Q\right]$$

Rearranging Equation (1) with Equation (2) and by substitution of the concentrations for fluorescence intensities yields:

(3) $$\frac{I_{0}}{I}\;=1+K_{ass}\left[Q\right]$$

where $I_{0}$ and I are the fluorescence intensities of the fluorophore in the absence and in the presence of the quencher (Q), respectively and $K_{ass}$ is the association constant for the formation of a non-fluorescent complex between the quencher and the fluorophore in the ground-state.

The graphical methods were performed at the wavelength of maximum intensity of the protein, 320 nm, using the computer program Origin 11 (OriginLab Corporation, Northampton, MA, USA).

#### 2.3.2. Surface Plasmon Resonance (SPR) Binding

SPR experiments were carried out using a Biacore X100 analytical system with a L1 sensor chip (GE Healthcare, Chicago, IL, USA), according to Sousa et al. [35]. Briefly, after immobilization of *E. coli* total lipid extract LUVs on channel 1 and of OmpF/*E. coli* total lipid extract proteoliposomes on channel 2, cycles of drug/compound injection were performed. Each cycle comprised injection of buffer, of drug/compound solution, association during 180 s, dissociation during 10 min and a final injection of buffer. Six concentrations (between 0 and 1 mmol dm^−3^) were tested for each studied drug/compound. The experiments were performed with liposomes concentration of 2 mmol dm^−3^ and proteoliposomes concentration of 2 µmol dm^−3^/2 mol dm^−3^ (OmpF/LUVs).

##### SPR Data Analysis

The equilibrium steady-state dissociation constants ($K_{d}$) were calculated through the fitting of 1:1 binding equation to the results, based on the values of R.U. (response units) at the equilibrium (t = 175 s) for each concentration used, as follows:

(4) $$R.U.\;=\frac{R.\;U._{max}x}{K_{d}+x}$$

The association constants ($K_{ass}$) were determined using the inverse of $K_{d}$:

(5) $$K_{ass}=\;\frac{1}{K_{d}}$$

### 2.4. Location Studies

The OmpF quenching studies were based on six Trp residues: three located at the interface bilayer/protein, near the phospholipid headgroups of the bilayer (Trp^214^—W214) and three close to the centre of the porin channel (Trp^61^—W61) [20,52]. The study of cpx and its metalloantibiotic with OmpF Trp (W61 and W214) was achieved in *E. coli* total extract proteoliposomes of two OmpF mutants (W61F and W214F), lacking one of each Trp, which is substituted by phenylalanine (Phe). The studies were performed with a maximum concentration of compound of 18 µmol dm^−3^ of cpx and of 5 µmol dm^−3^ of Cucpxphen. Additionally, fluorescence quenching of OmpF Trp residues (W61 and W214) by iodide and acrylamide were performed with *E. coli* total lipid extract proteoliposomes of native and mutant OmpF (W61F and W214F), in the absence and presence of compound solution. Small aliquots (µL) of quencher solution (iodide or acrylamide) were successively added to an *E. coli* total lipid extract/OmpF proteoliposome suspension of 1 mmol dm^−3^/1 µmol dm^−3^ (lipid/protein), in a concentration range from 0 to 0.5 µmol dm^−3^, in the absence or presence of cpx (≈30 µmol dm^−3^) or Cucpxphen (≈8 µmol dm^−3^) solution. After each addition, the mixture was equilibrated for five minutes and the spectra were traced, at 37.0 ± 0.1 °C, under constant stirring. The spectra were recorded with an excitation wavelength of 290 nm and emission wavelength range from 305 to 380 nm for WT and W61F and an excitation wavelength of 287 nm and emission wavelength range from 300 to 380 nm for W214F, an excitation slit width of 5 nm and an emission slit width of 10 nm, scan rate of 120 nm/minute and data range of 1 nm. Due to the lower excitation wavelength of W61 (W214F) proteoliposomes, emission filters of 295–1100 nm were applied in order to avoid the excitation of tyrosine [56]. All fluorescence experimental data were corrected for the dilution effect [53] and three independent measurements were performed.

#### Fluorescence Quenching Data Analysis

The collisional quenching phenomenon is described by the Stern-Volmer equation [56]:

(6) $$\frac{I_{0}}{I}\;=1+k_{q}\tau_{0}\left[Q\right]=1+K_{D}\left[Q\right]$$

where $I_{0}$ and I are the fluorescence intensities of the fluorophore in the absence and in the presence of the quencher (Q), respectively; $k_{q}$ is the bimolecular quenching constant; $\tau_{0}$ is the lifetime (average time that a molecule spends in the excited state) of the fluorophore in the absence of the quencher and [Q] is the concentration of the quencher. The Stern-Volmer quenching constant is given by $K_{D}=k_{q}\tau_{0}$.

In some cases, deviations to the linearity were observed in the Stern-Volmer plot of the quenching by iodide. When this plot exhibits a downward curvature, it is expected to exist two different populations of fluorophores with distinct accessibilities to the quencher. In this case, part of the total population is accessible to the quencher and other part is inaccessible [56]:

(7) $$I_{0}=I_{0a}+\;I_{0b}$$

where $I_{0}$ is the total fluorescence intensity of the protein, in the absence of the quencher; $I_{0a}$ and $I_{0b}$ are the fluorescence intensities of the accessible and inaccessible fractions, respectively.

In the presence of the quencher the fluorescence intensity observed (I) is given by:

(8) $$I\;=\frac{I_{0a}}{1+\;K_{a}\left[Q\right]}+\;I_{0b}$$

where $K_{a}$ is the Stern-Volmer constant of the accessible fraction and [Q] is the quencher concentration.

This modified form of the Stern-Volmer equation allows the calculation of the $K_{a}$ and of the accessible fraction $f_{a}$. The graphical linearization of the Equation (8) yields the following equation, that can be fitted to the experimental data:

(9) $$\frac{I_{0}}{\Delta I}\;=\frac{1}{f_{a}\;K_{a}\left[Q\right]}+\;\frac{1}{f_{a}}$$

where $f_{a}$ is the accessible fraction, given by $f_{a}=\frac{I_{0a}}{I_{0a}+I_{0b}}$.

The graphical methods were performed at the wavelength of maximum intensity of the protein (320 nm for WT, 318 nm for W214—mutant W61F—and 312 nm for W61—mutant W214F), using the computer program Origin 11 (OriginLab Corporation).

Acrylamide absorbs light at the excitation wavelength of the protein and both cpx and Cucpxphen solutions absorb light at the emission wavelength of the protein, under the used concentrations. The inner filter effect artifact was corrected using the equation described below [56,57,58]:

(10) $$I_{corr}=I_{obs}\;\times 10^{\left[\left(Abs_{ex}+Abs_{em}\right)/2\right]}$$

where $I_{corr}$ and $I_{obs}$ are the corrected and observed fluorescence intensities, respectively; $Abs_{ex}$ and $Abs_{em}$ are the absorbance values at the excitation and emission wavelengths used. Absorbance values take in account the path length of the absorption of the excitation and emission light of the cuvette.

## 3. Results and Discussion

### 3.1. Drug-Protein Interaction

During the last years, studies on the influx routes of FQs and their metalloantibiotics have been performed [35,36,37,38,39]. As previously mentioned, the Gram-negative cell wall is composed by different lipids, proteins and LPS. For this reason, the choice of a suitable mimetic system of Gram-negative bacterial membranes, comprising the lipid and protein components, is extremely important. Liposomes and proteoliposomes are usually used as membrane mimetic model systems [59,60,61]. In this study, proteoliposomes of OmpF were prepared using three different lipid compositions usually used to mimic *E. coli* membranes. Thus, this study begins with the evaluation of the importance of the lipid composition of the mimetic system on the association of FQs with OmpF.

#### OmpF-cpx Association in OmpF Proteoliposomes of POPE/POPG, POPE/POPG/CL and *E. coli* Total Extract

Cpx was chosen for the first experiments due to its OmpF-dependent translocation [23,35,37]. The interaction of cpx with OmpF was determined using three mimetic systems of *E. coli* membranes: the natural *E. coli* total extract, a binary mixture of POPE/POPG (0.75:0.25) and a ternary system of POPE/POPG/CL (0.67:0.23:0.10). These latter two mixtures are enriched with the major phospholipids of the *E. coli* membranes, being widely used as mimetic systems [36,62]. The success of the functional reconstitution of OmpF was previously confirmed according to Lopes et al. [47].

The presence of fluorescent residues in the structure of OmpF is responsible for its intrinsic fluorescence, which allows the use of fluorescence spectroscopy techniques to characterize the interactions between compounds and this porin. Besides Trp residues, the fluorescence of the proteins may also be due to other fluorescent amino acids as Tyr or Phe, especially if the absorption of the proteins occurs around 280 nm [56]. For this reason, the experiments were performed with an excitation wavelength of 290 nm, to assure that the observed fluorescence was only provided by the Trp residues.

**Figure 1:**
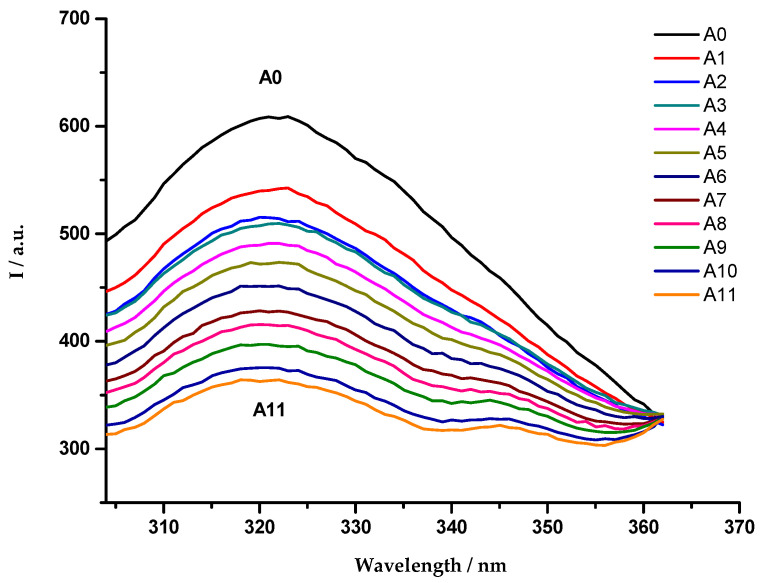
Emission fluorescence spectra of OmpF WT/*E. coli* total lipid extract proteoliposomes, in the absence (A0) and presence (A1–A11) of cpx solution (A1: 7.0 µmol dm^−3^; A2: 8.1 µmol dm^−3^; A3: 9.3 µmol dm^−3^; A4: 10.4 µmol dm^−3^; A5: 11.5 µmol dm^−3^; A6: 12.6 µmol dm^−3^; A7: 13.7 µmol dm^−3^; A8: 14.8 µmol dm^−3^; A9: 15.9 µmol dm^−3^; A10: 16.9 µmol dm^−3^; A11: 18.0 µmol dm^−3^), with excitation wavelength of 290 nm. Each spectrum is a mean of five replicate measurements.

The OmpF emission fluorescence spectra were recorded in the presence of increasing amounts of cpx solution, which resulted in a quenching of the OmpF fluorescence (Figure 1). This phenomenon was observed in the three studied systems.

**Figure 2:**
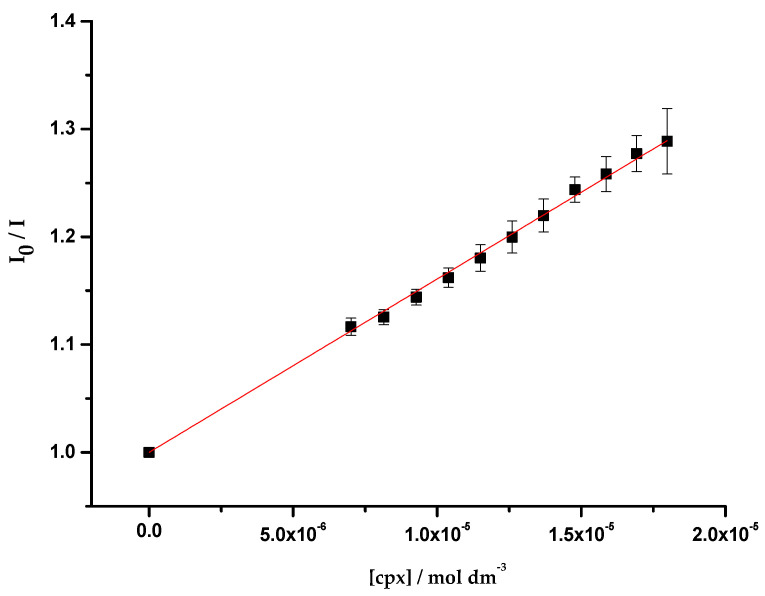
Plot of *I*_0_/*I* vs. cpx concentration ([cpx]), determined for OmpF WT/*E. coli* total extract proteoliposomes. Equation (3) was fitted to the experimental data. Data points are the mean of, at least, three independent experiments. Error bars are the SD.

As reported in previous studies, the experimental data revealed a static fluorescence quenching of the protein resultant from the presence of the antibiotics [54,55]. For this reason, and due to the linearity observed in the Stern-Volmer plot, the association constants ($K_{ass}$) were determined through the fitting of the Equation (3) to the quenching data (Figure 2).

The obtained association constants (Table 1) are analogous among the three studied systems, suggesting that the interaction of cpx with OmpF is independent of the lipidic composition of the environment. Furthermore, the $K_{ass}$ values obtained have the same magnitude of the values reported in the literature (log $K_{ass}$ cpx = 4.37 ± 0.05, determined with the porin in o-POE by fluorescence spectroscopy [55]; 3.15 < log $K_{ass}$ cpx < 4.00, determined in OmpF WT/DMPC proteoliposomes by fluorescence spectroscopy [63]; log $K_{ass}$ cpx = 3.90 ± 0.10, determined in OmpF WT/*E. coli* total extract proteoliposomes by SPR [35]).

According to the similarity of the experimental values, a representative mimetic system was chosen to proceed with the studies. Due to the complex composition but simplified preparation of the *E. coli* natural extract, OmpF WT/*E. coli* total extract proteoliposomes were adopted as the model membrane system of this study.

### 3.2. Association of FQs and Metalloantibiotics with OmpF

The work proceeded with a broad study of compound-OmpF interaction, through the assessment of the association of FQs and metalloantibiotics with OmpF, by fluorescence and SPR experiments.

#### 3.2.1. Fluorescence Studies

The interaction of five FQs and their respective metalloantibiotics with OmpF was assessed through the evaluation of the fluorescence of the Trp residues of the protein in the absence and presence of increasing amounts of each compound. The experiments were performed with *E. coli* total extract proteoliposomes of OmpF native protein (OmpF WT).

As described above, a static fluorescence quenching of the protein was observed, showing a linearity of the Stern-Volmer plot. Thus, the association constants ($K_{ass}$) were determined through the fitting of the Equation (3) to the quenching data (Table 2).

The $K_{ass}$ values obtained for the free FQs have the same magnitude of the values reported in the literature (log $K_{ass}$ mxfx = 4.65 ± 0.04, determined with the porin in o-POE by fluorescence spectroscopy [55]; log $K_{ass}$ spx = 3.74 ± 0.07, determined in OmpF WT/*E. coli* total extract proteoliposomes by SPR [35]). Furthermore, the values of the association constants obtained for free FQs and metalloantibiotics have the same magnitude.

The way in which FQs interact with porins has been subject of study to understand if there are preferential locations for their binding and to determine its magnitude. It had been observed that a stronger binding does not mean translocation and vice-versa. As an example, studies on the interaction of cpx and erx with OmpF and OmpC proved that cpx easily binds to OmpF and OmpC (in a similar way), while erx exhibited a stronger association to OmpF, although its structure is more hydrophobic [14]. However, besides the stronger interaction of erx, its uptake through OmpF is not easily achieved [20]. Another example is the association of mxfx to OmpF channel. Mxfx has a preferential interaction near the centre of the pore although it is known that this drug does not depend on the channel for its influx [23,52].

In turn, the $K_{ass}$ values determined for the metalloantibiotics are very similar to the ones of the free FQs, being even slightly higher. This same tendency was described in studies of the interaction of OmpF WT/*E. coli* total extract proteoliposomes with two metalloantibiotics (log $K_{ass}$ cpx = 3.90 ± 0.10 and log $K_{ass}$ Cucpxphen = 3.96 ± 0.08, and log $K_{ass}$ spx = 3.74 ± 0.07 and log $K_{ass}$ Cuspxphen = 4.15 ± 0.06, determined by SPR [35]). However, these experiments do not provide information about the specific region of the binding, not evidencing if the association occurs near or far from the constriction zone. Furthermore, Sousa et al. [35] stated a faster association and dissociation of Cucpxphen and Cuspxphen compared to their free FQs, meaning that a stronger association does not prove greater ability to cross the channel. Thus, we proceeded to the study of the binding of Cuerxphen, Cumxfxphen and Culvxphen to OmpF by SPR, to assess the steady-state binding affinity and the kinetics of the association and dissociation.

#### 3.2.2. SPR Binding

**Figure 3:**
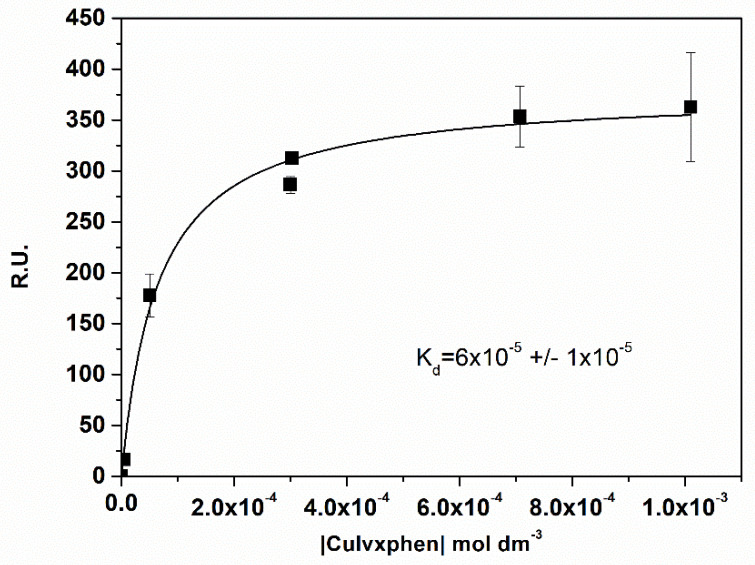
Plot of the Response Units (R.U.) at the equilibrium (t = 175 s) vs. the concentration of Culvxphen. $K_{d}$ was calculated with the non-linear fit of the one-site direct binding equation. Data points are a mean of, at least, three individual experiments. Error bars are the SD.

The equilibrium steady-state binding affinity was determined for three metalloantibiotics (Cuerxphen, Culvxphen and Cumxfxphen) by the inverse of the dissociation constants ($K_{d}$), obtained through the fitting of Equation (5) to the experimental data (Figure 3). The $K_{ass}$ values calculated (Table 3) are in agreement to the ones previously published by Sousa et al. [35]. Once again, a fast association/dissociation was observed for the metalloantibiotics, not allowing the calculation of the kinetic parameters.

Metalloantibiotics exist in solution predominantly in a mixture of two forms: mono-cationic ([Cu(FQ)phen]^+^) and di-cationic ([Cu(HFQ)phen]^2+^) forms. Therefore, the experimental results sustain a strong association of metalloantibiotics with the porin, possibly laying on electrostatic interactions with negative residues of the protein. Furthermore, the speciation of metalloantibiotics favours the diffusion across the membrane, as the di-cationic form privileges the electrostatic interactions with the negative surface of the bacterial membranes and the mono-cationic form behaves as a pseudo neutral form, as the copper charge is masked by the coordination with the phen and the FQ. Moreover, and analysing the size of the metalloantibiotics (~700–800 Da), it is expected a hampered translocation through channels that usually transport molecules up to 600 Da [9,64]. Thus, metalloantibiotics should adopt an influx route independent of porins, largely governed by electrostatic interactions with the membrane surface.

The experimental results obtained by fluorescence spectroscopy and SPR did not provide information about the region of the binding, as previously mentioned. However, theoretical experiments previously performed showed that the interaction of two metalloantibiotics (Cucpxphen and Cuspxphen) with OmpF should rely on residues located outside the constriction zone [35]. Thus, the work proceeded with a fluorescence approach to evaluate the preferential binding region of a free FQ and its metalloantibiotic with OmpF, through location studies.

### 3.3. Location Studies

OmpF is a homotrimeric protein whose monomers contain two Trp residues (W214 and W61) each. These aromatic residues are positioned in different regions of the channel: W214 is located close to the phospholipid headgroups, in the lipid-protein interface, in contrary to W61 that is placed in the centre of the channel [20,52]. Although with different location, both Trp are enclosed in hydrophobic environments, with a highest hydrophobicity neighbouring W61 [52,54]. Thereby, the study of the interaction of compounds with OmpF mutants (W61F and W214F), in which one of each Trp is substituted by Phe, may allow to infer about a preferential location for the binding. The mutant W214F provides information about the interaction of the drug near W61 (located in the centre of the bilayer), while W61F reveals the association near W214 (positioned at the interface of the bilayer/protein) [20,52]. Cpx and Cucpxphen were chosen for the first experiments since cpx is one of the vastly used FQs in the clinical practice. The $K_{ass}$ values determined are presented in Table 4.

The interaction of free cpx with W61 and W214 residues revealed differences. The $K_{ass}$ determined for cpx with OmpF W214F has a greater value (log $K_{ass}$ cpx = 4.24 ± 0.01) than the one obtained with the OmpF W61F (log $K_{ass}$ cpx = 4.13 ± 0.01), revealing a stronger association of cpx in the centre of the channel. Moreover, association with W214F has the same value of the previously determined in WT, showing that, in the presence of both Trp, cpx interacts preferentially with W61, near the centre of the channel. These results agree with previous studies that reported a privileged interaction of erx and mxfx close to W61, suggesting a preferential location of free FQs near the centre of the channel [20,52]. However, the studies previously performed by Fernandes et al. proposed a preferential binding of cpx with W214 (3.15 < log $K_{B}$ cpx < 3.62 to W61 and 3.58 < log $K_{B}$ cpx < 4.00 to W214, determined in OmpF WT/DMPC proteoliposomes by fluorescence spectroscopy) [63]. These differences may be due to the lipid composition of the systems. In this study we used proteoliposomes of *E. coli*, which is a negatively charged lipid system where cpx can partition (log $K_{p}$ cpx = 2.47 ± 0.03), contrarily to the zwitterionic DMPC, adopted by Fernandes et al., where there is no ability of cpx to partition [36]. Thus, the constants determined in DMPC do not account with interactions between cpx and liposomes, which may explain reduced values and the enhanced association with Trp residues of the protein located near the headgroups of the *E. coli* phospholipids.

In contrast, the $K_{ass}$ values determined for Cucpxphen with the OmpF mutant (W61F and W214F) proteoliposomes exhibit analogous values for both Trp residues. These results show a similar interaction of the metalloantibiotic with the residues located near the centre of the channel (W214F) or in the bilayer/protein interface (W61F).

The experimental data revealed differences in the fluorescence intensity and in the wavelength of maximum intensity of the proteoliposomes (320 nm for WT, 318 nm for W61F and 312 nm for W214F). The lower the emission wavelength of the Trp, the higher the hydrophobicity of the surrounding environment [56]. Besides the lower wavelength of maximum intensity, the mutant W214F exhibited the higher fluorescence intensity, indicative of a greater hydrophobicity of the surrounding environment of W61, as previously stated by other authors [52,54].

The work proceeded with the study of the quenching of OmpF by iodide and acrylamide. These two molecules provide information about different regions of the membrane, complementing the location studies performed in this section.

#### Fluorescence Quenching of OmpF by Iodide and Acrylamide

Due to their chemical differences, iodide and acrylamide are able to quench Trp residues located in different regions of the membrane [56]. Iodide is negatively charged, providing information about the Trp residues located in more hydrophilic environments, in the protein/membrane interface (W214), while the polar behaviour and the neutral charge of the acrylamide favour the quenching of the Trp residues positioned in environments with higher hydrophobicity, near the centre of the pore (W61) [20,52,56]. Therefore, these studies may provide information about the location of drugs according to the accessibility of the Trp residues to the quenchers [56].

**Figure 4:**
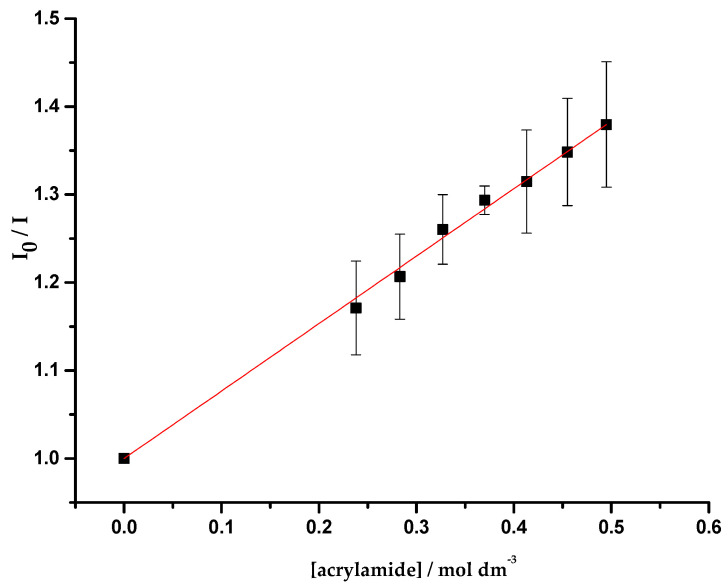
Plot of *I*_0_/*I* vs. acrylamide concentration ([acrylamide]), determined for the quenching of OmpF WT/*E. coli* total lipid extract liposomes by acrylamide, with excitation and emission wavelengths of 290 and 320 nm, respectively. Equation (6) was fitted to the experimental data. Data points are the mean of, at least, three independent experiments. Error bars are the SD.

**Figure 5:**
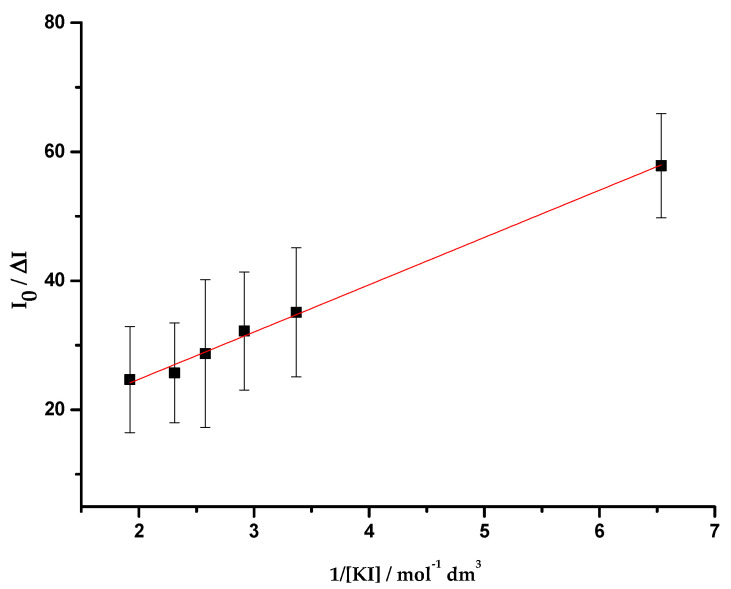
Plot of *I*_0_/Δ*I* vs. the inverse of the iodide concentration (1/[KI]), determined for the quenching of OmpF WT/*E. coli* total lipid extract liposomes by iodide, in the presence of Cucpxphen solution, with excitation and emission wavelengths of 290 and 320 nm, respectively. Equation (9) was fitted to the experimental data. Data points are the mean of, at least, three independent experiments. Error bars are the SD.

This part of the work comprised quenching studies of OmpF (WT, W61F and W214F)/*E. coli* total extract proteoliposomes by iodide and acrylamide, in the absence and presence of cpx and Cucpxphen. The experimental data exhibited linear Stern-Volmer plots (Figure 4), except for the quenching of OmpF WT/*E. coli* total extract proteoliposomes by iodide in the presence of the metalloantibiotic (Figure 5). The linear Stern-Volmer plots suggest that all Trp residues have similar accessibility to the quencher. The quenching constants, determined by the fitting of the Equations (6) and (9) to the experimental data, are summarized in Table 5.

The results obtained for the quenching of W214F proteoliposomes by acrylamide show a decrease of the Stern-Volmer constant ($K_{D}$) in the presence of cpx, suggesting that cpx shields the W61 from the action of the quencher. This outcome supports a location of cpx near the centre of the porin, as proposed by the experimental results of the previous section. These results reveal a behaviour of cpx similar to the one previously reported for erx in the mutant D113N, by Mahendran et al. [20]. In contrast, the $K_{D}$ values determined for the quenching of W61F proteoliposomes by acrylamide are analogous in the absence and presence of cpx, suggesting that is not expected to find cpx near W214. The fact that acrylamide can quench W214 proves that the environment enclosing these residues is more hydrophobic than it was expected. Hence, the results obtained for the quenching of WT proteoliposomes by acrylamide may provide information about both Trp.

In the presence of Cucpxphen, the $K_{D}$ value obtained for the quenching of W214F proteoliposomes by acrylamide increases, revealing that W61 is more exposed to the quencher in the presence of the metalloantibiotic. The exposure may arise from conformational changes resultant from the binding of Cucpxphen in other regions of the protein.

The quenching of the proteoliposomes by iodide was only observed in WT proteoliposomes in the presence of Cucpxphen. In this case, the Stern-Volmer plot exhibited a negative deviation from the linearity (Figure 5), revealing the existence of two populations of Trp residues with different accessibility to the quencher: one accessible and other not accessible. Since Trp residues accessible to iodide must be faced to the surrounding medium (in the surface of the protein) [56], it means that there are two different populations of W214, one unreachable to iodide (around 90% of the residues) and the other (about 10%) exposed to it. The fact that no quenching was observed in WT proteoliposomes in the absence of both compounds demonstrates that W214 is more embedded in the membrane than what was expected, being inaccessible to iodide. Combining these results, it is possible to infer that W214, unreachable to iodide, becomes exposed in the presence of Cucpxphen, probably due to conformational modifications in the protein.

Taking together, the location results suggest a preferential interaction of cpx near the centre of the porin (with W61), which supports a hydrophilic pathway (OmpF dependent) for the influx of this FQ. These results corroborate previous studies that stated that OmpF facilitates the translocation of cpx [23,35,37]. Concerning Cucpxphen, this metalloantibiotic showed no preferential interaction among W61 and W214. Nevertheless, its presence gave rise to an enhanced exposure of both Trp to the quenchers, probably due to conformational changes in the porin. These outcomes support a hydrophobic pathway (independent of the porin or using the porin/bilayer interface) for the influx of Cucpxphen.

The biophysical outcomes obtained in this work corroborate the knowledge previously achieved by microbiological and theoretical experiments [23,35], evidencing the relevance of combining multidisciplinary approaches for the understanding of the influx pathways of drugs.

## 4. Conclusions

The reduction of the intracellular concentration of drugs is one of the main bacterial resistance mechanisms developed against antibiotics. Metalloantibiotics of FQ/Cu(II)/phen seem a potential alternative to free FQs based on an influx route independent of porins, especially OmpF and OmpC.

In this work, we studied the permeation of FQs and its metalloantibiotics in *E. coli*, evaluating the role of OmpF, the major porin involved in the uptake of FQs in *E. coli*, for its influx.

Biophysical studies were carried out to determine the interaction of the compounds with OmpF. Three mimetic systems were initially used (proteoliposomes of OmpF inserted in POPE/POPG, POPE/POPG/CL and natural total extract of *E. coli*), showing comparable interaction for cpx with OmpF among the three systems. Therefore, OmpF WT/*E. coli* total extract proteoliposomes were chosen as the bacterial membrane model of this study.

The association constants assessed for pure FQs and metalloantibiotics with OmpF WT, by fluorescence spectroscopy and SPR, were similar. Nevertheless, the assays performed with mutants of OmpF and quenchers (iodide and acrylamide) showed differences in the interaction of cpx and its metalloantibiotic with the porin. The pure cpx exhibited a preferential interaction near the centre of the pore, revealing a specific binding with the porin, while Cucpxphen revealed a similar association in different regions of the channel (both in the centre as in the periphery).

These results corroborate previous studies [23,25,30,43,44] that propose that metalloantibiotics may be a promising substitute to FQs against *E. coli*, due to its presumable ability to circumvent the AMR mechanism based on the reduction of the expression of porins. Furthermore, toxicological data show that metalloantibiotics are non-toxic at MIC concentrations (data not shown). Therefore, the suitability of these metalloantibiotics against *E. coli* resistant strains should be assessed through microbiological experiments, focusing on multidrug-resistant clinical isolates with known antibiotic resistance profiles revealing decreased membrane permeability. The possible use of these metalloantibiotics may improve the costs and time inherent to the drug development processes.

## Figures and Tables

**Table 1:** Values of the association constants ($K_{ass}$) of cpx with OmpF proteoliposomes of POPE/POPG, POPE/POPG/CL and E. coli total lipid extract, obtained by steady-state fluorescence, using Equation (3). The presented values are the mean of, at least, three independent experiments (±SD).

Lipid Model System	$\mathrm{Log}\;K_{ass}$
POPE/POPG	4.31 ± 0.01
POPE/POPG/CL	4.27 ± 0.01
*E. coli* total lipid extract	4.21 ± 0.01

**Table 2:** Values of the association constants ($K_{ass}$) of OmpF-compounds, determined with OmpF WT/E. coli total extract proteoliposomes, by steady-state fluorescence, using Equation (3). The presented values are the mean of, at least, three independent experiments (±SD).

Porin	Antibiotic	$\mathrm{Log}\;K_{ass}$	
		FQ	CuFQphen
OmpF WT	cpx	4.21 ± 0.01 ^a^	4.56 ± 0.01
	erx	4.37 ± 0.01	4.40 ± 0.01
	lvx	4.53 ± 0.01	4.62 ± 0.01
	mxfx	4.63 ± 0.01	4.73 ± 0.01
	spx	4.47 ± 0.01	4.58 ± 0.01

^a^ Value determined in the previous section.

**Table 3:** Values of the association constants ($K_{ass}$) of OmpF-compounds, determined with OmpF WT/E. coli total extract proteoliposomes, by SPR, using Equation (5). The presented values are the mean of, at least, three independent experiments (±SD).

Porin	CuFQphen	$\mathrm{Log}\;K_{ass}$
OmpF WT	Cuerxphen	4.00 ± 0.09
	Culvxphen	4.22 ± 0.07
	Cumxfxphen	4.30 ± 0.09

**Table 4:** Values of the association constants ($K_{ass}$) of OmpF-compounds, determined with OmpF (W61F and W214F)/E. coli total extract proteoliposomes, by steady-state fluorescence, using Equation (3). The presented values are the mean of, at least, three independent experiments (±SD).

Porin	$\mathrm{Log}\;K_{ass}$	
	Cpx	Cucpxphen
OmpF W214F	4.24 ± 0.01	4.47 ± 0.02
OmpF W61F	4.13 ± 0.01	4.47 ± 0.01
OmpF WT	4.21 ± 0.01 ^a^	4.56 ± 0.01 ^a^

^a^ Value determined in the previous section.

**Table 5:** Values of the Stern-Volmer constants ($K_{D}$ and $K_{a}$) determined for the quenching of OmpF (WT, W61F and W214F)/E. coli total extract proteoliposomes by iodide and acrylamide, in the absence and in the presence of cpx and Cucpxphen solutions, by steady-state fluorescence, using Equations (6) and (9). The presented values are the mean of, at least, three independent experiments (±SD).

Porin	Compound	$K_{D}/{\mathrm{mol}}^{-1}\;{\mathrm{dm}}^{3}$			
		Compound Absent		Compound Present	
		Acrylamide	Iodide	Acrylamide	Iodide
OmpF W214F	cpx	0.49 ± 0.01	N.d.	0.32 ± 0.01	N.d.
	Cucpxphen		N.d.	1.43 ± 0.02	N.d.
OmpF W61F	cpx	0.54 ± 0.02	N.d.	0.57 ± 0.01	N.d.
	Cucpxphen		N.d.	N.d.	N.d.
OmpF WT	cpx	0.77 ± 0.01	N.d.	0.77 ± 0.01	N.d.
	Cucpxphen		N.d.	N.d.	$K_{a}$ = 1.38 ± 0.01; f_a_ = 0.10 ^a^

^a^$K_{a}$ obtained by the fitting of the Equation (9) to the experimental data.
